# Comparison of Different Chronic Maintenance Antithrombotic Strategies in Patients with Coronary Artery Disease: A Systematic Review and Network Meta-Analysis

**DOI:** 10.1155/2023/5446271

**Published:** 2023-08-17

**Authors:** Junyan Zhang, Zhongxiu Chen, Yujia Cai, Chen Li, Yong He

**Affiliations:** ^1^Department of Cardiology, West China Hospital of Sichuan University, Chengdu, China; ^2^Chinese Evidence-Based Medicine Center and MAGIC-China Center, West China Hospital, Sichuan University, Chengdu, China

## Abstract

**Background:**

Optimal antithrombotic therapy during the chronic maintenance period in patients with coronary artery disease (CAD) is unknown. We compared five kinds of mainstream chronic maintenance antithrombotic strategies at least one year after the acute phase: aspirin alone, clopidogrel alone, ticagrelor alone, continued dual antiplatelet therapy (DAPT) for a period of time, and maintenance with aspirin combined with a low-dose anticoagulant such as rivaroxaban.

**Methods:**

Ten randomized, controlled trials were selected using PubMed, Ovid MEDLINE, Embase, and Cochrane library through February 2023. The primary outcome was main adverse cardiac events (MACEs), and secondary outcomes include net adverse clinical events (NACEs), cardiac death, all-cause death, ischemic stroke, stent thrombosis, total bleeding, and major bleeding. A network meta-analysis was conducted with a random-effects model. Data extraction was performed by three independent reviewers.

**Results:**

Our search identified ten eligible randomized controlled trials enrolling a total of 82,084 patients comparing different chronic maintenance antithrombotic strategies. As for the primary endpoint, there was no statistical difference in MACE outcomes between any two of the five methods. As for the secondary endpoint, there was no statistical difference in NACE, major bleeding, all-cause death, cardiac death, and stent thrombosis between any two methods. The aspirin plus low-dose rivaroxaban group had a lower incidence of ischemic stroke compared to the aspirin group (OR = 0.49, 95% CrI 0.26-0.91). And the prolonged DAPT group had a higher total bleeding rate compared to aspirin group (OR = 2.4, 95% CrI 1.1-5.9).

**Conclusions:**

In terms of MACE, NACE, all-cause death, cardiac death, stent thrombosis, and major bleeding, there were no significant differences between using aspirin alone, clopidogrel alone, and ticagrelor alone; extending DAPT duration; and using aspirin combined with low-dose rivaroxaban for chronic maintenance antithrombotic regimens. However, choosing aspirin combined with low-dose rivaroxaban can reduce the incidence of ischemic stroke, and prolonged DAPT may have a higher rate of total bleeding. However, it is important to note that this study is based on indirect comparisons, and there is currently a lack of direct evidence comparing various maintenance antiplatelet therapy regimens. Further high-quality studies are needed to address this gap and provide more conclusive evidence on the comparative effectiveness of different maintenance antiplatelet strategies.

## 1. Introduction

Patients with coronary artery disease (CAD) typically require lifelong treatment with a single antiplatelet medication, often aspirin, as a secondary prevention strategy during the chronic maintenance phase to reduce the risk of ischemic events. Aspirin is a nonselective cyclooxygenase inhibitor, a classic drug that has undergone extensive testing in clinical studies, with ample evidence supporting its clear benefits for secondary prevention of cardiovascular disease. It is recommended in numerous guidelines, including the 2018 ESC/EACTS guidelines on myocardial revascularization, which recommend lifelong single antiplatelet therapy, usually aspirin [1]. The main rationale for recommending aspirin comes from earlier collaborative analyses on aspirin alone, several decades ago, rather than from comparative studies of aspirin versus clopidogrel [2, 3].

However, recent studies, such as HOST-EXtended Antiplatelet Monotherapy (HOST-EXAM) and its extended study, have begun to identify the superiority of clopidogrel over conventional aspirin in secondary prevention [4, 5]. At the same time, several studies suggest that extending dual antiplatelet therapy (DAPT) beyond 12 months is more beneficial in high-risk populations, due to the high ischemic nature of acute coronary syndrome (ACS). The collaborative meta-analysis by Udell et al. [6] demonstrated that prolonging the duration of DAPT significantly reduces ischemic events such as cardiovascular death, recurrent myocardial infarction, and stroke in ACS population.

Moreover, a subgroup analysis of the COMPASS study demonstrated the feasibility of using a combination of low-dose rivaroxaban and aspirin during the chronic maintenance phase of CAD [7]. Despite the increased risk of major bleeding with the addition of rivaroxaban to aspirin, it lowered the incidence of major adverse cardiac events (MACEs) as well as net adverse clinical events (NACEs). Based on the subgroup analysis results of the COMPASS trial, both the 2019 European Society of Cardiology (ESC) guidelines for the management and diagnosis of chronic coronary artery syndrome (CCS) [8] and the 2020 ESC guidelines for the management of non-ST-segment elevation acute coronary syndromes (NSTE-ACS) [9] recommend, in agreement, that for high-risk CAD patients without a high risk of bleeding, consideration should be given to adding a second antiplatelet drug, such as low-dose rivaroxaban 2.5 mg bid, on top of aspirin for long-term secondary prevention (IIa, A).

Therefore, various studies have proposed different antithrombotic options for the chronic maintenance phase of CAD, including maintenance with aspirin alone, clopidogrel alone, and ticagrelor alone; continuation of DAPT for a period of time followed by monotherapy; and maintenance with aspirin combined with low-dose rivaroxaban. However, the optimal antithrombotic strategy for patients with CAD during chronic maintenance remains unclear. This study utilized a trial-level network meta-analysis to compare the safety and efficacy of the five different maintenance treatment strategies.

## 2. Methods

### 2.1. Search Strategy

This meta-analysis was performed in compliance with the Cochrane Intervention Review that compares multiple interventions and the network meta-analysis extension for the Preferred Reporting Items for Systematic Reviews and Meta-Analyses (PRISMA-NMA) guidelines. At the outset, the study protocol was registered with PROSPERO (ID: CRD42022382564). We included randomized controlled trials (RCTs) that examined the impact of various chronic maintenance antithrombotic strategies for patients with CAD. Our search strategy was systematic, and we looked for relevant studies from the beginning of each database until February 1, 2023, by using keywords such as “coronary artery disease,” “CAD,” “percutaneous coronary intervention,” “PCI,” “antiplatelet therapy,” “DAPT,” “antithrombotic therapy,” “prospective,” “randomized control trial,” and “RCT” in PubMed, Embase, Ovid MEDLINE, and the Cochrane Library. Additionally, we conducted a manual search of secondary sources, including the references of initially identified articles, reviews, and commentaries [10]. We downloaded all references for consolidation, elimination of duplicates, and further analyses. No language restrictions were applied.

### 2.2. Study Selection

Several assessments were performed, followed by the removal of duplicate articles after the initial screening. The titles and abstracts of relevant publications were further screened for suitability before full article retrieval. Additionally, meeting abstracts, editorials, and reviews were also checked and excluded from the analysis. We included studies that met the following criteria: (1) compared antithrombotic regimens in patients with CAD in the chronic maintenance phase, defined as more than one year after PCI surgery or more than one year after the occurrence of an ACS event; (2) were published in peer-reviewed journals with full texts available; and (3) reported cardiovascular clinical outcomes. We excluded trials in which monotherapy is initiated after a short period of DAPT (e.g., 1 month, 3 months, or 6 months). Two investigators (JYZ and ZXC) independently reviewed all retrieved studies, and differences were resolved via consensus.

### 2.3. Data Extraction and Quality Assessment

Study data, including the first author's name, research name, publish year, study type, sample size, maintenance regimen, clinical baseline characteristics, mean follow-up duration, types of major adverse cardiac events (MACE), types of net adverse clinical events (NACE), and other outcomes were independently extracted by three investigators (JYZ, ZXC, and CL). The study quality was independently evaluated by two cardiologists (JYZ and ZXC) according to the revised Cochrane risk-of-bias tool for randomized trials (ROB2) [11]. Disagreements were again resolved by consulting a third reviewer.

### 2.4. Study Outcome

The primary outcome was MACE, which mostly included all-cause death, ischemic stroke, myocardial infarction, and stent thrombosis. Although, there are slight variations in the definition of MACE among different studies. We used the definitions provided by each individual study as the standard. Secondary outcomes included NACE, cardiac death, all-cause death, stent thrombosis, total bleeding, major bleeding, and ischemic stroke. NACE mostly included all-cause death, ischemic stroke, myocardial infarction, stent thrombosis, and major bleeding. We followed the definitions of outcomes used in each trial.

### 2.5. Statistical Analysis

The R software (version 4.1.2, R Foundation for Statistical Computing, Beijing, China) was used to perform a network meta-analysis of various interventions. The analysis was conducted using the “gemtc 1.0-1” and “rjags 4-13” packages, employing a Bayesian network framework with a Monte Carlo Markov Chain (MCMC) model and consistency model. Four MCMC chains were run simultaneously for 100,000 iterations with 50,000 simulations. Model convergence was evaluated using tract and density plots and the Brooks-Gelman-Rubin diagnosis plot. A high degree of convergence was determined if each MCMC chain in the track had an overlapping area that covered most of the chain fluctuation range, the density graph was normally distributed, and the bandwidth approached zero and remained stable. The consistency of direct and indirect comparisons was analyzed using the node splitting test, and a *P* value ≥ 0.05 indicated satisfactory consistency. The overall heterogeneity of the model was assessed using the “anohe” function to calculate the deviation of the size of the heterogeneity variance parameter *I*^2^. The advantages and disadvantages of interventions were measured using the surface under the cumulative probability ranking (SUCRA). Unlike traditional frequentist statistics, this study employed a Bayesian approach in conducting the network meta-analysis. Therefore, the interval estimates in this study are presented as 95% credible interval (CrI).

## 3. Results

### 3.1. Study Selection

**Figure 1:**
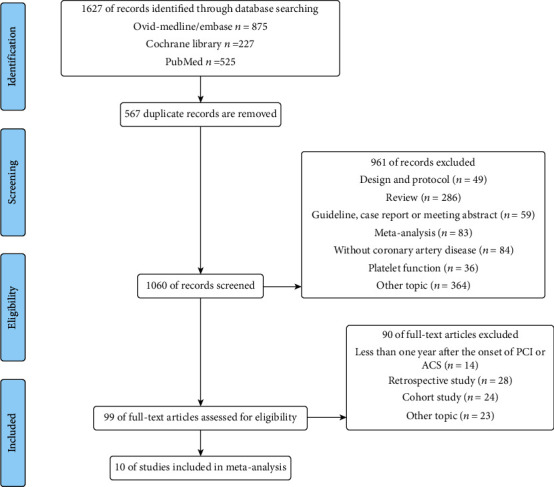
PRISMA flow diagram of the study selection. PRISMA: Preferred Reporting Items for Systematic Reviews and Meta-Analyses statement.

We identified 525 publications in PubMed, 227 publications in the Cochrane Library, and 875 publications in Embase and Ovid MEDLINE combined. Of these 1627 studies, 567 were found to be duplicates. Ten of the remaining studies met the inclusion criteria. Details of the search strategy are shown in Figure 1.

### 3.2. Study Characteristics and Quality Assessment

All ten included studies were RCTs without any cohort studies. Two of the included studies compared aspirin with clopidogrel for chronic maintenance stage [4, 12], while two compared aspirin with ticagrelor [13, 14], five compared aspirin with prolonged DAPT [15–19], and one compared aspirin with aspirin plus low-dose rivaroxaban [7]. Meanwhile, all nine studies reported MACE, eight reported NACE, eight reported major bleeding, and seven reported in-stent thrombosis. For the quality assessment of RCTs, the scale mainly included the following: (1) generation of random sequence (selection bias), (2) concealment of distribution sequence (selection bias), (3) blind method for research object and implementer (implementation bias), (4) blind method for result evaluation (measurement bias), (5) incomplete result (loss of follow-up bias), (6) selective report (report bias), and (7) other bias. All included studies were high quality. The participants varied in age from 60.3 to 80.3 years. The percentages of patients who were male sex and who had diabetes, hypertension, dyslipidemia, and previous MI ranged from 69.1%–81.0%, 14.0%–100%, 38.0%–92.8%, 41%–88.8%, and 3.7%–100%, respectively. The general characteristics of patients and outcome events in the included studies are summarized in Tables 1 and 2.

### 3.3. Convergence, Consistency, and Heterogeneity Analysis

**Figure 2:**
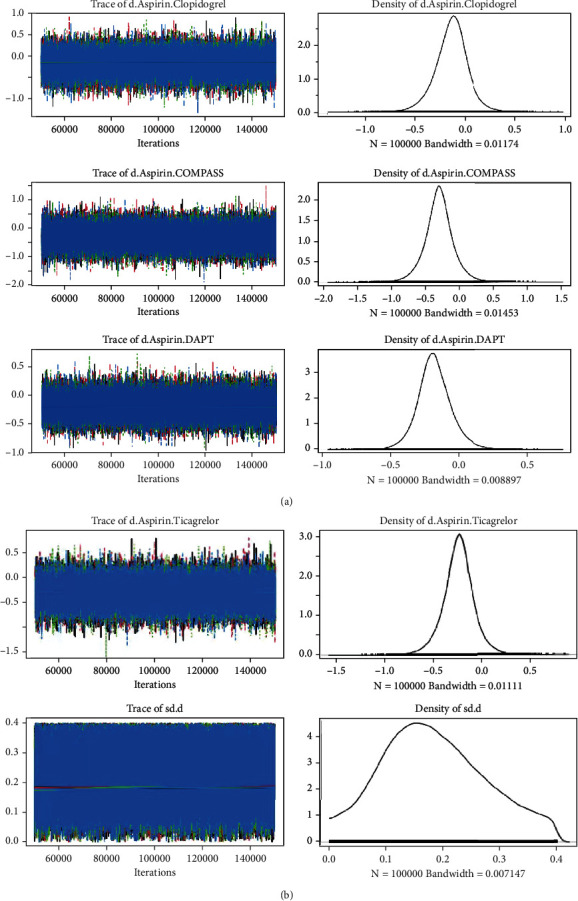
The trace and density plot and Brooks-Gelman-Rubin diagnosis plot. DAPT: dual antiplatelet therapy.

**Figure 3:**
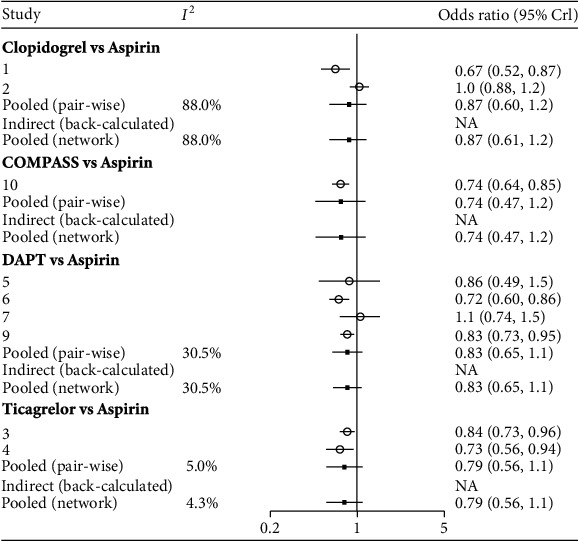
The forest diagram of heterogeneity analysis. DAPT: dual antiplatelet therapy.

The convergence of the models was assessed by the trace and density plot (Figure 2) and Brooks-Gelman-Rubin diagnosis plot (Figure 2). The naked eye cannot recognize single chain fluctuation, and the density map was normally distributed, suggesting an excellent convergence of these models. This study did not form a closed loop, so there is no need to compare the differences between direct and indirect calculations. The heterogeneity analysis suggested a heterogeneity that exists in the comparison of aspirin and clopidogrel (*I*^2^ = 0.88); no other heterogeneity was discovered in the overall pooled analysis (Figure 3).

### 3.4. Risk of MACE and NACE in Different Chronic Maintenance Treatment Strategies

**Figure 4:**
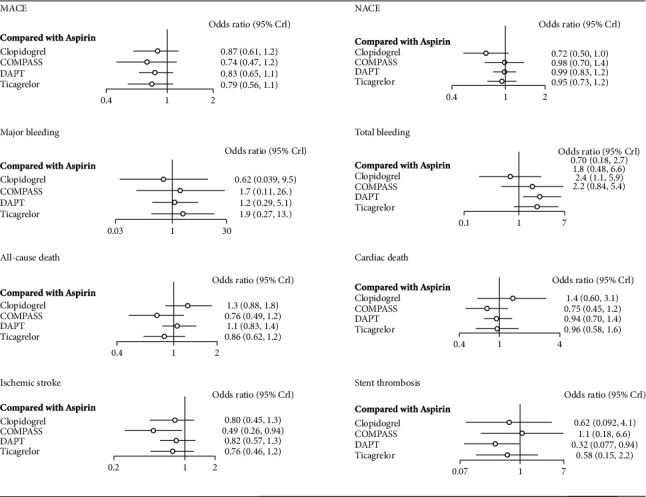
Forest plot: MACE, NACE, major bleeding, total bleeding, all-cause death, cardiac death, ischemic stroke, and stent thrombosis; COMPASS: aspirin plus low-dose rivaroxaban; DAPT: dual antiplatelet therapy; OR: odds ratio; MACE: main adverse cardiac event; NACE: net adverse clinical event. All comparisons are with aspirin monotherapy.

**Figure 5:**
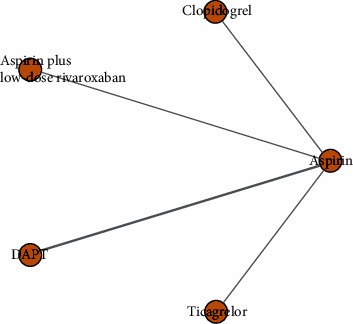
Network plot of available direct comparisons for primary outcome MACE. Each circle represents a chronic maintenance antiplatelet strategy. Solid lines connect treatment options that have been directly compared, and the thickness of the lines is proportional to the number of studies included. MACE: main adverse clinical outcome.

A total of nine studies containing five interventional arms (aspirin, clopidogrel, ticagrelor, prolonged DAPT, and aspirin plus low-dose rivaroxaban) analyzed the endpoint of MACE. The definition of MACE in different studies depends on the individual study and mostly includes all-cause death, myocardial infarction, ischemic stroke, and in-stent thrombosis. The pooled synthesis results of commutative comparisons between specific treatments are displayed in Figure 2, measured by OR and its 95% CrI. There was no significant increase in MACE in all remaining groups (clopidogrel, ticagrelor, prolonged DAPT, and aspirin plus low-dose rivaroxaban) when compared with the aspirin group (Figure 4) (OR and 95% CrI: 0.87, 0.61 to 1.2; 0.79, 0.56 to 1.1; 0.83, 0.65 to 1.1; 0.74, 0.47 to 1.2). To further explore the hierarchy of curative effect in those therapies, we established a histogram of ranking probability (Figure 4). For the NACE outcome, we likewise found no significant reduction in NACE events with the remaining four regimens (clopidogrel, ticagrelor, prolonged DAPT, and aspirin plus low-dose rivaroxaban) compared to aspirin (OR and 95% CrI: 0.72, 0.50 to 1.1; 0.95, 0.74 to 1.2; 0.96, 0.81 to 1.2; 0.98, 0.71 to 1.4). The above data showed no significant differences in MACE and NACE outcomes corresponding to the five different chronic maintenance antithrombotic regimens. Interestingly, clopidogrel had a tendency to have lower NACE events compared to other regimens. The network plot for the primary outcome MACE is provided in Figure 5.

### 3.5. Risk of All-Cause Death and Cardiac Death in Different Chronic Maintenance Treatment Strategies

For the endpoint of all-cause death, all nine studies were included. The results suggest that other four maintenance antithrombotic regimens for CAD also failed to reduce all-cause mortality and cardiovascular mortality compared with aspirin. Regarding all-cause mortality, the OR and 95% CrI for each of the four treatment strategies were as follows: 1.3 (0.88-1.8) for clopidogrel, 0.86 (0.62-1.2) for ticagrelor, 1.0 (0.78-1.3) for DAPT, and 0.76 (0.48-1.2) for aspirin plus low-dose rivaroxaban (Figure 4). As for cardiac death, the OR and 95% CrI for each of the four treatment strategies were as follows: clopidogrel 1.4 (0.6, 3.1), ticagrelor 0.96 (0.58, 1.6), DAPT 0.91 (0.69, 1.3), and aspirin plus low-dose rivaroxaban 0.75 (0.45, 1.2) (Figure 4).

### 3.6. Risk of Major Bleeding, In-Stent Thrombosis, Ischemic Stroke, and Total Bleeding in Different Chronic Maintenance Treatment Strategies

In terms of major bleeding, the four other regimens showed no significant trend toward lower bleeding compared with aspirin, with ORs and 95% CrIs of 0.62 (0.039, 9.5) for clopidogrel, 1.9 (0.27, 13.0) for ticagrelor, 1.2 (0.29, 5.1) for DAPT, and 1.7 (0.11, 26.0) for aspirin plus low-dose rivaroxaban (Figure 4). In terms of in-stent thrombosis, the incidence was also similar in other four groups compared with the aspirin group, with ORs and 95% CrIs of 0.62 (0.076, 5.0) for clopidogrel, 5.8 (0.13, 2.5) for ticagrelor, 0.41 (0.13, 1.5) for DAPT, and 1.1 (0.14, 8.1) for aspirin plus low-dose rivaroxaban (Figure 4). In terms of ischemic stroke, the incidence was lower in the aspirin plus low-dose rivaroxaban group than that in the aspirin group, OR = 0.49, 95% CrI 0.26-0.91 (Figure 4). And prolonged DAPT had a higher total bleeding rate compared to aspirin monotherapy (OR = 2.4, 95% CrI 1.1-5.9).

## 4. Discussion

Navarese et al. [20] recently conducted a network meta-analysis comparing nine different antithrombotic regimens for maintenance treatment in patients with CAD at 12 months. The results suggested that P2Y_12_ monotherapy can reduce the risk of myocardial infarction, while aspirin combined with 2.5 mg rivaroxaban can reduce the probability of stroke. However, the study included significant heterogeneity and several regimens, such as aspirin combined with VKA, 5 mg rivaroxaban monotherapy, and placebo monotherapy, which are currently rarely used in clinical practice. To address this issue, we conducted a more precise network meta-analysis by including studies with stronger homogeneity and utilizing the five most mainstream antithrombotic regimens for maintenance treatment.

This network meta-analysis examines chronic maintenance antithrombotic strategies for CAD patients at least one year after the acute phase. The analysis includes ten well-designed randomized control trials (RCTs) and finds that, while various antithrombotic strategies during the chronic maintenance period show similar incidences of MACE and NACE compared to aspirin, aspirin plus low-dose rivaroxaban is associated with a lower incidence of ischemic stroke compared to aspirin alone. No significant differences are observed in the incidence of all-cause death, cardiac death, in-stent thrombosis, and major bleeding among the various antithrombotic strategies during the chronic maintenance phase for CAD patients.

Regardless of whether CAD patients receive reperfusion therapy such as PCI, in the chronic maintenance period after the acute phase, current guidelines recommend that patients receive long-term antiplatelet monotherapy for a long time or even lifelong maintenance treatment [21]. As a secondary prevention strategy, long-term maintenance of antiplatelet therapy can prevent ischemic events such as reinfarction, stent thrombosis, and other events from occurring. Aspirin has been the standard therapy for chronic maintenance treatment of CAD for decades, according to several guidelines. It is the most widely used antiplatelet agent because it has the longest history of use and the strongest clinical evidence of efficacy and safety. As the body of evidence continues to grow, recent studies appear to be challenging the longstanding status of aspirin.

### 4.1. Aspirin versus Clopidogrel

The CAPRIE study published in 1996 suggested that clopidogrel may have potential benefits in atherosclerotic cardiovascular disease, such as reduction in cardiovascular events [12]. However, since the era of drug-eluting stents (DES), there have been no randomized trials for a long time that clearly indicate which is better, aspirin or clopidogrel, for long-term antiplatelet monotherapy. This question was first tested in the recently published HOST-EXAM study, which included 5530 patients who entered chronic maintenance after PCI and were randomized to the clopidogrel and aspirin groups and followed for two years [4]. Not only was the incidence of ischemic events significantly lower in the clopidogrel group than in the aspirin group (3.7% vs. 5.5%, HR = 0.68, 95% CrI: 0.52-0.87, *P* = 0.0028); the risk of bleeding (Academic Research Consortium (BARC) bleeding ≥ type 2) was also significantly lower in the clopidogrel group compared with the aspirin group (2.3% vs. 3.3%, HR = 0.70, 95% CrI. 0.51 to 0.98, *P* = 0.036). The subsequently published HOST-EXAM Extended study continued to extend the follow-up to 5.8 years, concluding that clopidogrel alone significantly reduced the risk of NACE by 26% compared with aspirin, with both efficacy and safety benefits [5]. In this network meta-analysis, although there was no statistically significant difference between aspirin and clopidogrel in terms of primary and secondary endpoints, clopidogrel showed a trend toward superiority over several other regimens in terms of NACE outcomes.

### 4.2. Aspirin versus Ticagrelor

Ono et al. reported the results of a landmark analysis of the GLOBAL LEADERS trial [13]. The study included 5308 cases treated with ticagrelor monotherapy 1 year after PCI, as well as 5813 cases treated with aspirin monotherapy. In the second year after PCI, the ischemic composite endpoint was reduced by 26% with ticagrelor monotherapy compared with aspirin monotherapy (1.9% vs. 2.6%: log-rank *P* = 0.014, adjusted HR 0.74, 95% CI: 0.58-0.96; *P* = 0.022), primarily due to a reduced risk of myocardial infarction. In contrast, bleeding was increased in bleeding BARC type 3 or 5 with ticagrelor monotherapy (0.5% vs. 0.3%: log-rank *P* = 0.051, adjusted HR 1.89, 95% CI: 1.03-3.45; *P* = 0.005). This study adds valuable information comparing ticagrelor and aspirin monotherapy in the long-term maintenance period after PCI. In fact, The GLOBAL LEADERS trial was originally designed in 2013, before the clinical value of a lower dose of ticagrelor (60 mg bid) had been established for chronic maintenance treatment. As a result, using ticagrelor 60 mg bid in the trial may have produced different results than those obtained today. In this study, it is possible that ticagrelor did not demonstrate a significant superiority compared to other treatment options, likely due to the 90 mg dose of ticagrelor reducing the risk of ischemia in patients while simultaneously increasing the risk of bleeding, resulting in no significant net clinical benefit. Further research is necessary, with a focus on comparing the effects of 60 mg ticagrelor during the chronic maintenance period.

### 4.3. Aspirin vs. Prolonged DAPT

Up to the present time, large RCTs have compared the differences between DAPT longer than 24 months and standard DAPT duration. Although the conclusions of these studies are not entirely consistent, they all show a basic perspective; that is, prolonging DAPT duration can reduce the occurrence of major ischemic adverse events such as myocardial infarction and in-stent thrombosis, but at the cost of increased bleeding events, which is consistent with the conclusion of this network meta-analysis [15–17]. As the newer generation DESs greatly reduce the risk of stent thrombosis due to their thinner stent struts, better biocompatible polymers, and good drug elution properties, there is currently a trend towards shorter term DAPT strategies. However, for some patients with extremely high ischemic risk and low bleeding risk, we can still consider appropriately prolonging DAPT to combat these high ischemic risks. Howard et al. [22] summarized the clinical characteristics that can benefit from prolonging DAPT duration, including ACS, diabetes, peripheral vascular disease, smoking, and left ventricular ejection fraction less than 30%, among others. Nevertheless, overall, clinicians need to balance the ischemic and bleeding risks of patients to choose a more personalized and precise DAPT duration for them.

### 4.4. Aspirin versus Aspirin plus Low-Dose Rivaroxaban

This network meta-analysis suggests that the use of aspirin combined with low-dose rivaroxaban as a chronic maintenance regimen reduces the incidence of ischemic stroke events by 51% compared with aspirin alone. This finding was most likely driven by the CAD subgroup of the COMPASS study included in this study [7]. In the CAD subgroup of the COMPASS study, the incidence of ischemic stroke was twice as high in the aspirin monotherapy group as in the aspirin combined with low-dose rivaroxaban group (2% vs. 1%). In fact, the COMPASS study was the first to demonstrate the feasibility of aspirin in combination with low-dose rivaroxaban in patients with CCS [23]. This regimen reduced the composite risk of stroke, cardiac death, and reinfarction in patients with CAD and peripheral artery disease by 24%, reduced the relative risk of stroke by 42%, and reduced all-cause mortality by 18%, despite an increased risk of major bleeding in patients. Afterwards, in 2019, the ESC included this treatment strategy in the CCS guidelines and recommended it as a class IIA recommendation for patients with high ischemic risk and low bleeding risk [8]. Overall, aspirin combined with 2.5 mg rivaroxaban is a treatment option with great promise, and with more research and practical experience, we believe that this treatment option will be more widely used in future clinical practice.

### 4.5. Individualized Antithrombotic Strategy: An Inevitable Trend

Although this study did not identify any significant differences among five different chronic antiplatelet therapy regimens with respect to the primary endpoint, it offers us a wider range of feasible options for selecting the optimal antiplatelet therapy. By evaluating the ischemic and bleeding risks of patients, we can confidently customize the selection of different maintenance antiplatelet regimens. For instance, ticagrelor or prolonged DAPT can be employed for patients at high risk of ischemia, while aspirin combined with low-dose rivaroxaban can be chosen for those at high risk of stroke. For patients at high risk of bleeding, less bleeding-risky monotherapy with clopidogrel can be a preferred option. Nevertheless, more evidence is required to support these regimens. We look forward to future studies on maintenance antiplatelet therapy for high-risk ischemic, bleeding, and even patients who have both a high risk of ischemia and a high risk of bleeding, which can more accurately evaluate the optimal antiplatelet regimens.

## 5. Limitations

This study had several limitations. Firstly, this study indirectly compared several maintenance antithrombotic regimens other than aspirin using a network meta-analysis method, while a direct comparison was lacking. Secondly, it should be noted that although this study enrolled a relatively homogeneous population, studies such as THEMIS-PCI only included patients with diabetes, which represent high ischemic risk. Future research may need to pay greater attention to chronic maintenance treatment strategies for patients at high-risk ischemia or high risk of bleeding. Thirdly, this study is a study-level network meta-analysis, and it is important to acknowledge that study-level analyses have inherent limitations when it comes to recognizing and addressing study heterogeneity.

## 6. Conclusion

This study compared five different antithrombotic strategies for chronic maintenance period in CAD and found no significant differences in terms of MACE, NACE, all-cause death, cardiovascular death, major bleeding, and stent thrombosis events between any of the pairwise comparisons. Aspirin combined with low-dose rivaroxaban was found to better reduce the incidence of ischemic stroke compared to standard strategy aspirin. Prolonged DAPT may lead to more total bleeding compared to aspirin. Clopidogrel showed a trend of lower NACE compared to other regimens. However, this study has limitations such as the lack of direct comparison between different antithrombotic strategies and that more clinical research is needed to directly compare the differences among various chronic maintenance antithrombotic strategies in order to better guide clinical practice. All in all, each patient requires precise and individualized antithrombotic strategies for chronic maintenance period to achieve a better balance between bleeding and ischemic risks.

## Figures and Tables

**Table 1:** Baseline characteristics of the included studies.

Research/year	Maintenance regimen	Age, yr.	Male, *n* (%)	ACS, *n* (%)	Hypertension, *n* (%)	Diabetes, *n* (%)	Dyslipidemia, *n* (%)	Chronic kidney disease, *n* (%)	Previous MI, *n* (%)
HOST-EXAM trial/2021	Aspirin (*n* = 2728)	63·4 (10·7)	2039 (74·7%)	1957 (71.7%)	1674 (61·4%)	935 (34·3%)	1883 (69·0%)	337 (12·4%)	435 (15·9%)
	Clopidogrel (*n* = 2710)	63·5 (10·7)	2015 (74·4%)	1964 (72.5%)	1664 (61·4%)	925 (34·1%)	1884 (69·5%)	356 (13·1%)	437 (16·1%)

CAPRIE/1996	Aspirin (*n* = 3159)	58·3 (11·3)	81%	34%	38%	15%	42%	ND	ND
	Clopidogrel (*n* = 3143)	58·6 (11·4)	81%	34%	39%	14%	41%	ND	ND

THEMIS-PCI/2019	Aspirin (*n* = 5596)	66·0 (61·0–72·0)	3880 (69.8)	ND	5136 (91·8)	5596 (100%)	4967 (88·8)	ND	ND
	Ticagrelor (*n* = 5558)	66·0 (61·0–72·0)	3838 (69.1)	ND	5127 (92·2)	5558 (100%)	4922 (88·6)	ND	ND

GLOBAL LEADERS landmark-analysis/2022	Aspirin (*n* = 5813)	64.1 ± 10.0	4519 (77.7)	2585 (44.5%)	72.8 (4,214/5,792)	24.1 (1,402/5,810)	70.4 (3,970/5,639)	ND	22.9 (1,326/5,801)
	Ticagrelor (*n* = 5308)	63.7 ± 10.2	4135 (77.9)	2566 (48.3%)	73.4 (3,885/5,290)	24.3 (1,287/5,303)	69.6 (3,561/5,119)	ND	21.8 (1,153/5,295)

ARCTIC-Interruption/2014	Aspirin (*n* = 624)	64 (57–73)	503 (81%)	ND	388 (62%)	222 (36%)	426 (68%)	ND	186 (30%)
	Aspirin plus clopidogrel (*n* = 635)	64 (57–73)	508 (80%)	ND	376 (59%)	198 (31%)	428 (67%)	ND	197 (31%)

DAPT/2014	Aspirin (*n* = 4941)	61.6 ± 10.1	3657 (74%)	2103 (42.6%)	3649/4934 (74.0)	1481/4927 (30.1)	ND	ND	1026/4870 (21.1)
	Aspirin plus clopidogrel (*n* = 5020)	61.8 ± 10.2	3778 (75.3%)	2148 (42.8%)	3796/5006 (75.8)	1556/5006 (31.1)	ND	ND	1092/4953 (22.0)

DES-LATE/2013	Aspirin (*n* = 2514)	62.3 ± 10.1	1749 (69.6%)	1551 (61.7%)	1423 (56.6%)	709 (28.2%)	ND	ND	92 (3.7%)
	Aspirin plus clopidogrel (*n* = 2531)	62.5 ± 10.0	1749 (69.1%)	1512 (59.7%)	1479 (58.4%)	709 (28.0%)	ND	ND	103 (4.1%)

OPTIDUAL/2016	Aspirin (*n* = 690)	64.2 + 11.5	547 (79.3%)	262 (38.0%)	417 (60.4)	222 (32.2)	ND	ND	122 (17.7)
	Aspirin plus clopidogrel (*n* = 695)	64.1 + 10.8	568 (81.7%)	239 (34.4%)	396 (57.0)	213 (30.6)	ND	ND	119 (17.1)

PEGASUS-TIMI 54/2015	Aspirin (*n* = 7067)	65.4 ± 8.3	5350 (75.7%)	6652/7057 (94.3%)	5484 (77.6)	2257 (31.9)	5451 (77.1)	1649/6985 (23.6)	7067 (100%)
	Aspirin plus ticagrelor 60 mg (*n* = 7045)	65.2 ± 8.4	5384 (76.4%)	6599/7035 (93.8%)	5461 (77.5)	2308 (32.8)	5380 (76.4)	1547/6955 (22.2)	7045 (100%)
	Aspirin plus ticagrelor 90 mg (*n* = 7050)	65.4 ± 8.4	5368 (76.1%)	6661/7043 (94.6%)	5462 (77.5)	2241 (31.8)	5410 (76.7)	1653/6958 (23.8)	7050 (100%)

COMPASS/2017	Aspirin (*n* = 8261)	69 (65–73)	6615 (80%)	ND	6280 (76%)	3040 (37%)	ND	ND	5721 (69%)
	Aspirin plus low-dose rivaroxaban (*n* = 8313)	69 (65–73)	6577 (79%)	ND	6218 (75%)	3043 (37%)	ND	ND	5654 (68%)

BMI: body mass index; PCI: percutaneous coronary intervention; RCT: randomized controlled trial; STEMI: ST-segment elevation myocardial infarction; ACS: acute coronary syndrome; DAPT: dual antiplatelet therapy; MI: myocardial infarction; ST: stent thrombosis; MACE: major adverse cardiac event; NACE: net adverse clinical event.

**Table 2:** Clinical outcome of the included studies.

Research/year	Maintenance regimen	NACE		MACE		All-cause death		Cardiac death		Ischemic stroke		Major bleeding		Stent thrombosis	
		Event	Total	Event	Total	Event	Total	Event	Total	Event	Total	Event	Total	Event	Total
HOST-EXAM trial/2021	Aspirin (*n* = 2728)	207	2728	146	2728	36	2728	14	2728	26	2728	53	2728	16	2728
	Clopidogrel (*n* = 2710)	152	2710	99	2710	51	2710	19	2710	14	2710	33	2710	10	2710

CAPRIE/1996	Aspirin (*n* = 3159)	ND	5843	283	5843	97	5843	ND	5843	42	5843	ND	5843	ND	5843
	Clopidogrel (*n* = 3143)	ND	5787	291	5787	111	5787	ND	5787	42	5787	ND	5787	ND	5787

THEMIS-PCI/2019	Aspirin (*n* = 5596)	581	5596	480	5596	323	5596	183	5596	113	5596	101	5596	18	5596
	Ticagrelor (*n* = 5558)	601	5558	404	5558	282	5558	174	5558	88	5558	197	5558	9	5558

GLOBAL LEADERS landmark-analysis/2022	Aspirin (*n* = 5813)	310	5813	151	5813	66	5813	ND	5813	26	5813	17	5813	16	5813
	Ticagrelor (*n* = 5308)	236	5308	101	5308	51	5308	ND	5308	17	5308	28	5308	10	5308

ARCTIC-Interruption/2014	Aspirin (*n* = 624)	28	624	27	624	9	624	ND	624	4	624	1	624	3	624
	Aspirin plus clopidogrel (*n* = 635)	30	635	24	635	7	635	ND	635	6	635	7	635	0	635

DAPT/2014	Aspirin (*n* = 4941)	357	4941	285	4941	74	4941	47	4941	34	4941	72	494	65	4941
	Aspirin plus clopidogrel (*n* = 5020)	340	5020	211	5020	98	5020	45	5020	24	5020	129	5020	19	5020

DES-LATE/2013	Aspirin (*n* = 2514)	74	2514	57	2514	32	2514	19	2514	13	2514	24	2514	11	2514
	Aspirin plus clopidogrel (*n* = 2531)	89	2531	61	2531	46	2531	28	2531	15	2531	34	2531	7	2531

OPTIDUAL/2016	Aspirin (*n* = 690)	52	690	ND	690	24	690	14	690	4	690	14	690	1	690
	Aspirin plus clopidogrel (*n* = 695)	40	695	ND	695	16	695	10	695	4	695	14	695	3	695

PEGASUS-TIMI 54/2015	Aspirin (*n* = 7067)	632	7067	578	7067	326	7067	210	7067	103	7067	54	7067	ND	7067
	Aspirin plus ticagrelor 60 mg (*n* = 7045)	602	7045	487	7045	289	7045	174	7045	78	7045	115	7045	ND	7045
	Aspirin plus ticagrelor 90 mg (*n* = 7050)	620	7050	493	7050	326	7050	182	7050	88	7050	127	7050	ND	7050

COMPASS/2015	Aspirin (*n* = 8261)	618	8261	460	8261	339	8261	184	8261	120	8261	158	8261	46	8261
	Aspirin plus low-dose rivaroxaban (*n* = 8313)	610	8313	347	8313	262	8313	139	8313	60	8313	263	8313	50	8313

MACE: major adverse cardiac event; NACE: net adverse clinical event.

## Data Availability

The data supporting this meta-analysis are from previously reported studies and datasets, which have been cited. The processed data are available from the corresponding authors upon request.
